# Impairment of the GABAergic system in the anterior insular cortex of heroin-addicted males

**DOI:** 10.1007/s00406-024-01848-2

**Published:** 2024-07-09

**Authors:** Anna Gos, Johann Steiner, Kurt Trübner, Christian Mawrin, Michał Kaliszan, Tomasz Gos

**Affiliations:** 1 Department of Adult Psychiatry and Psychotherapy, Psychiatric University Hospital, Zurich, Switzerland; 2 Department of Psychiatry, Otto von Guericke University, Magdeburg, Germany; 3 Institute of Legal Medicine, University of Duisburg-Essen, Essen, Germany; 4 Department of Neuropathology, Otto von Guericke University, Magdeburg, Germany; 5 Department of Forensic Medicine, Medical University of Gdańsk, Ul. Dębowa 23, 80-204 Gdańsk, Poland

**Keywords:** Heroin addiction, Anterior insular cortex, GAD 65/67 immunostaining

## Abstract

Opioid addiction is a global problem, causing the greatest health burden among drug use disorders, with opioid overdose deaths topping the statistics of fatal overdoses. The multifunctional anterior insular cortex (AIC) is involved in inhibitory control, which is severely impaired in opioid addiction. GABAergic interneurons shape the output of the AIC, where abnormalities have been reported in individuals addicted to opioids. In these neurons, glutamate decarboxylase (GAD) with its isoforms GAD 65 and 67 is a key enzyme in the synthesis of GABA, and research data point to a dysregulation of GABAergic activity in the AIC in opioid addiction. Our study, which was performed on paraffin-embedded brains from the Magdeburg Brain Bank, aimed to investigate abnormalities in the GABAergic function of the AIC in opioid addiction by densitometric evaluation of GAD 65/67-immunostained neuropil. The study showed bilaterally increased neuropil density in layers III and V in 13 male heroin-addicted males compared to 12 healthy controls, with significant U-test *P* values for layer V bilaterally. Analysis of confounding variables showed that age, brain volume and duration of formalin fixation did not confound the results. Our findings suggest a dysregulation of GABAergic activity in the AIC in opioid addiction, which is consistent with experimental data from animal models and human neuroimaging studies.

**Supplementary Information:**

The online version contains supplementary material available at 10.1007/s00406-024-01848-2.

## Introduction

Opioid addiction is a serious global problem, as opioids are consistently the drug group causing the greatest health harm in terms of deaths and disability-adjusted life years, and remain the leading cause of overdose deaths according to the annual World Drug Report [1].

The insular cortex (IC), located at the base of the lateral sulcus, is extensively connected to cortical areas and subcortical regions involved in the regulation of homeostasis and behaviour, and plays numerous sophisticated functional roles. The most specific of these seems to be the interoception of internal bodily states, their integration into conscious feelings and their incorporation into cognitive and motivational processes [2–4]. The aversive interoceptive stimuli experienced during withdrawal, and the memory of them, are crucial for perpetuating the withdrawal-craving-intoxication vicious cycle in addiction [5]. However, the prominent role of IC dysfunction in this cycle is not limited to the withdrawal phase, as supported by experimental and clinical reports (for reviews see: [2, 6, 7]). Accordingly, the IC has recently emerged as a promising target for brain stimulation as a novel therapeutic option for addiction [7].

The more posterior, granular regions of the insula, which receive input from the thalamus and parietal, occipital and temporal association cortices, are involved in somatosensory, vestibular and motor integration. These granular regions provide information flow to more anterior, agranular regions that are involved in the integration of autonomic and visceral information and are reciprocally connected to limbic regions of the prefrontal cortex, amygdala and nucleus accumbens, among others [3, 8, 9]. Thus, the anterior insular cortex (AIC) is involved in neuronal networks that control decision making and execution, which are profoundly distorted in addiction [10].

GABAergic dysfunction has been implicated in addiction (for reviews see: [11, 12]). Gamma-aminobutyric acid (GABA) is the main inhibitory neurotransmitter in the mature brain, and glutamic acid decarboxylase (GAD), with GAD 65 and 67 isoforms, is the rate-limiting enzyme involved in converting glutamate to GABA. Transiently activated GAD 65 appears to be restricted to membranes and nerve terminals. Constitutively active GAD 67 is more widely distributed in neurons and plays a major role in GABAergic function [13, 14]. GABAergic neurotransmission is critical for synaptic plasticity [15], neurogenesis [16] and synchronous neuronal oscillations [17]. Abnormalities in these fundamental processes, involving dysfunction of GABAergic neurons, have been suggested in addiction by experimental studies in animal models and human data [18–20]. To date, reports of GABAergic abnormalities in the insula are sparse compared to other components of the addiction circuitry, and human postmortem studies of the GABAergic system in the IC in addiction are lacking. However, existing experimental data from animal models suggest that dysfunction of GABAergic interneurons in the IC may be involved in the reinforcement of opioid addiction [21–23].

Recently, we found a reduced volume of the AIC in heroin-addicted males who died from heroin overdose [24]. We therefore hypothesised that this volumetric abnormality might be accompanied by an impairment of the GABAergic system, as our group has found in the globus pallidus [25, 26]. We tested this hypothesis by using GAD 65/67 immunostaining in paraffin-embedded brain sections and quantitative evaluation of the relative density of GAD 65/67-immunoreactive (GAD-ir) neuropil in the AIC of the previously studied cohort. We focused on neuropil density because this morphological marker corresponds better than somata density to disturbed function of GABAergic interneurons at the synaptic level and thus to disturbed inhibitory neurotransmission, as suggested by experimental research in animal models of brain disorders [27, 28] and our previous research in depression and schizophrenia [29–31].

## Material and methods

### Characteristics of the subjects

All brains were obtained from the Magdeburg Brain Bank, where they were provided by one of the authors (K.T.) from medico-legal autopsies at the Institute of Legal Medicine, University of Duisburg-Essen. Sampling and preservation of the human brain material were done in accordance with the Declaration of Helsinki, German law and the local institutional review board at the University of Magdeburg. The analysis included 13 heroin-addicted males who died suddenly from heroin overdose and 12 male control cases of sudden natural death from cardiopulmonary arrest (the detailed diagnostic and demographic data of the cases examined are shown in the Supplementary Table). The sample of brains from heroin overdose victims was collected over a longer period of time because such cases are rare. Therefore, the fixation time of these brains was much longer than that of the control brains. Information on clinical characteristics was extracted from the available clinical records and through structured interviews with persons in close contact to the heroin-addicted individuals. According to the available information, the tested heroin overdose victims exclusively met the diagnostic criteria for heroin addiction, even though they occasionally used other substances (see Supplementary Table: heroin-addicted individuals, substances used in addition to heroin). An experienced neuropathologist (C.M.) found no evidence of neurodegenerative diseases (such as Alzheimer’s, Parkinson’s, Pick’s disease), tumors, inflammatory, vascular or traumatic processes or hypoxic neuronal damage, using sections with Nissl-myelin staining and HLA-DR, beta-amyloid, and tau immunostaining to evaluate cortical regions (including the prefrontal areas and the insular cortex), the hippocampal formation, the basal ganglia and the brainstem. None of heroin-addicted individuals was HIV-positive. A toxicological analysis of blood and urine for heroin, ethanol, and other substances of abuse was performed at each medico-legal autopsy as a part of forensic postmortem diagnostics. The cause of death in all heroin-addicted individuals was established by an experienced forensic pathologist (K.T.).

### Tissue preparation and immunohistochemistry

After embedding in paraffin, serial 20-µm thick coronal sections were cut along the rostrocaudal axis of the cerebral hemispheres and then mounted. Each 25th section was deparaffinised, rehydrated and stained with a combined cell and fiber staining according to Nissl (cresyl violet) and Heidenhain-Woelcke (myelin). Volume shrinkage was determined after dehydration and embedding of tissue using the formula: VSF = (A1/A2)^3/2^ (VSF = volume shrinkage factor, A1 = cross-sectional area before processing and A2 = cross-sectional area after processing of tissue; the value of power of 3/2 means that the square root of the surface shrinkage factor, which determines the linear shrinkage factor, was then raised to the third power, which in turn determines the volume shrinkage factor). The mean volume shrinkage factor was 2.02 for controls and 1.80 for heroin-addicted individuals (a non-significant *t*-test *P* value = 0.20 for the comparison between both groups).

**Fig. 1:**
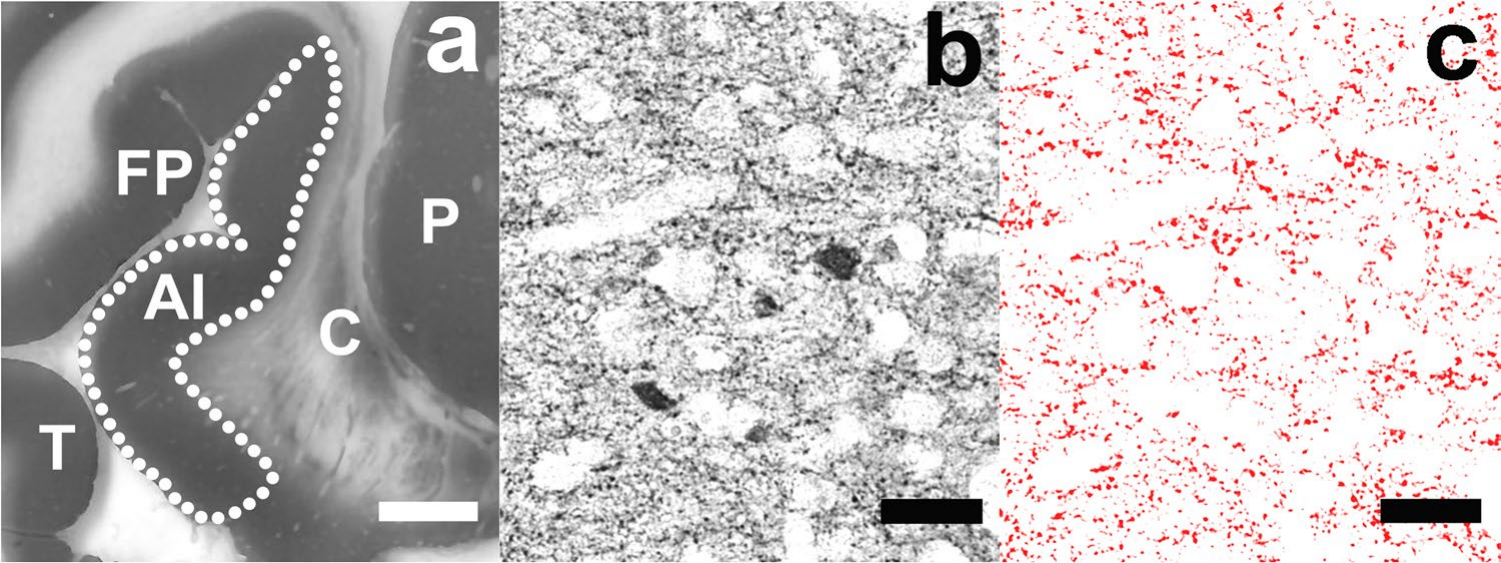
**a** Low**-**magnification image of the fragment of the GAD 65/67-immunostained coronal section of the right brain hemisphere of the heroin-addicted individual at the level at which the investigations were carried out (*AI* anterior insular cortex, *C* claustrum, *FP* frontoparietal operculum, *P* putamen, *T* temporal operculum, scale bar 5 mm) **b** Appearance of GAD 65/67 immunostaining, which was similar in AI layers III and V bilaterally and in both groups compared. Thus, differences in neuropil density between heroin-addicted individuals and controls could not be assessed qualitatively, but could be measured quantitatively (the right layer V of the case shown at **a**, scale bar 50 μm) **c** Same area as in **b**, where the GAD 65/67-immunoreactive neuropil marked by the CellSens computer program was selected for automatic calculation of its relative density after exclusion of the neuronal bodies (scale bar 50 μm)

Stained coronal sections of the brains were taken at the level where the AIC was visible, i.e. the part of the insular region anterior to the central insular sulcus, delineated by the anterior, superior, and inferior periinsular sulci according to the established neuroanatomical criteria [32] (Fig. 1a). From the Nissl-myelin stained sections in which the AIC was visible along the antero-posterior dimension, two were randomly selected, starting from the most anterior one close to the anterior periinsular sulcus. The sections adjacent to them were stained with a mouse anti-human GAD 65/67 monoclonal antibody (Medical & Biological Laboratories Co., Woburn, USA; product number M018-3, clone 9A6, subclass mouse IgG1 kappa). The specificity of the antibody has been confirmed by the supplier using Western blotting and immunohistochemistry. Therefore, two immunostained sections at the level of the AIC were used to evaluate the relative GAD-ir neuropil density in cortical layers III and V bilaterally in each of the cases studied. Nissl-myelin stained sections adjacent to the immunostained sections were used to identify the cortical layers.

Formalin-fixed tissue sections were deparaffinised and antigen retrieval was performed by boiling the sections for 4 min in 10 mM citrate buffer (pH 6.0). Sections were preincubated with methanol/H_2_O_2_ to block endogenous peroxidase activity. After repeated washing with phosphate-buffered saline, the primary GAD-specific antibody was applied for 72 h at 4 °C (dilution 1:100). The washing process was repeated and sections were incubated with biotinylated anti-mouse secondary antibody (dilution 1:100) for 2 h at room temperature (Amersham Biosciences, Ltd., Little Chalfont, UK). After washing, sections were incubated with 1:100 streptavidin–biotin-peroxidase complex for 1 h at room temperature (Amersham Biosciences), 3,3’-diaminobenzidine was used for peroxidase visualisation and 0.5% ammonium nickel sulphate hexahydrate was applied to enhance the signal (see Fig. 1b for the staining pattern). Negative control sections without primary antibody showed no immunostaining.

### Quantification

Images were acquired with an Olympus BX60 microscope equipped with a DP22 digital camera and CellSens Dimension Desktop v1.18 software using a 20 × objective. Quantitative morphological analysis was performed in each of the selected sections as previously reported [29–31]. The relative density of GAD-ir neuropil (the quotient of neuropil area and total sampling box area, see below) of heroin-addicted individuals and controls was measured bilaterally in the AIC layers III and V. Three randomly selected boxes were approached in each of four ROIs (i.e., no specific selection criteria were applied). The number of boxes required to obtain reliable data was determined by statistical analysis of previous data, with ten boxes per structure evaluated bilaterally [29]. The area of the sampling boxes was 0.340 ± 0.040 mm^2^ (this method does not require the application of a constant measuring field).

To measure the area of immunostained structures, the immunoreactive neuropil was visualised by manually adjusting the minimum and maximum grey levels of the nickel-enhanced DAB precipitate under visual control. Thresholds were adjusted separately for each section to minimise the bias associated with the staining procedure (i.e. where background staining was very intense, the threshold was down-regulated, and where it was very weak, it was up-regulated to obtain the most reliable automatic labelling of neuronal structures). After labelling and exclusion of the labelled neuronal somata, all remaining red-labelled elements representing the neuropil were applied for further calculation, independently of labelling intensity (see Fig. 1c). The area of the labelled immunoreactive neuropil was automatically calculated and divided by the total area of the sampling box to give the relative area of immunoreactive neuropil (in percent). The aim was to detect the difference in GABAergic neuropil density between the compared groups according to the method described previously [29–31], rather than to obtain absolute values of the stained neuropil density.

Measurements were performed by one of the authors (A.G.) who was blinded to diagnosis. Repeat measurements were performed on 5 brains to determine inter-rater (A.G., T.G.) and test–retest reliability. Intraclass correlation analyses yielded correlation coefficients ranging from 0.90 to 1.00 for both inter-rater and test–retest reliability.

### Data analysis

Statistical analyses were performed using the data analysis software system STATISTICA version 10 (StatSoft®, Inc. 2011, www.statsoft.com). As the analysed GAD-ir neuropil density was not normally distributed (i.e. significant values of the Kolmogorov and Lilliefors tests were obtained in the compared groups), non-parametric statistical procedures in hierarchical mode were used.

First, the generalised linear/nonlinear models (GLZ) module of STATISTICA with the general custom designs (GCD) procedure was used as an omnibus method to analyse associations between the dependent variable (i.e. GAD-ir neuropil density) and independent categorical variables (i.e. heroin addiction/control groups, layer, and side). The results of the GCD analysis were automatically reported, including the Wald statistic value, degrees of freedom, and the corresponding *P* value. Age, postmortem interval, brain volume and fixation time were considered as numerical confounding variables. Spearman correlation coefficients were calculated to determine the impact of these variables which might confound GAD-ir neuropil density. These coefficients were also calculated to determine correlations between GAD-ir neuropil densities and AIC volumes found previously [24].

Subsequently to GCD analysis, unadjusted two-way *post-hoc* comparisons with Mann–Whitney *U*-test were used to detect possible differences between the study groups with respect to the variables mentioned above (i.e. GAD-ir neuropil density and confounders). All statistical tests were two-tailed. Generally, *P* values of < 0.05 were accepted as statistically significant.

## Results

### Quantitative analysis of the GAD 65/67 immunostained neuropil

Differences in the GAD-ir neuropil density in the AIC between heroin-addicted individuals and controls could not be assessed qualitatively, but could be captured by quantitative measurements, as previously reported by our group in other cortical regions in depression [29, 30] and schizophrenia [31].

The initial omnibus analysis of the results of all 4 investigated ROIs (i.e. from the bilaterally evaluated layers III and V, 52 mean values from heroin-addicted individuals and 48 mean values from controls) by the GCD procedure revealed a significant effect of the diagnosis (*P* = 0.000754) without associations with the investigated cortical layer and the investigated hemisphere. Consistent with this initial analysis, further ROI-specific analyses using *post-hoc U*-tests revealed increases in the target parameter in heroin-addicted individuals in all four ROIs, which were significant in layer V bilaterally (see Table 1 and Supplementary Table).

**Table 1:** Results of between-group comparisons of the relative density (in percent) of glutamic acid decarboxylase immunoreactive (GAD-ir) neuropil in the layers III and V of the anterior insular cortex

ROI and group	GAD-ir neuropil relative density [%] Median (q1, q3, n)	*U*-test* P* H/C
Layer III left		0.110
H	2.50 (1.73, 2.83, 13)	
C	1.59 (1.42, 2.09, 12)	
Layer III right		0.077
H	2.38 (2.23, 3.40, 13)	
C	1.79 (1.55, 2.67, 12)	
Layer V left		**0.022**
H	2.53 (2.04, 3.12, 13)	
C	1.73 (1.34, 2.08, 12)	
Layer V right		**0.035**
H	2.77 (2.48, 2.92, 13)	
C	2.12 (1.64, 2.40, 12)	

*ROI* region of interest, *H* heroin-addicted individuals, *C* controls, *q1 and q3* quartile 1 and 3, *n* number of cases, *U-test P* U-test *P* value (significant values are in bold)

### Confounders

Age, brain volume and fixation time revealed significant differences between heroin-addicted individuals and controls (significant *U*-test *P* values, see Table 2 A and Supplementary Table). However, correlation analysis did not suggest that significant differences in GAD-ir neuropil density observed bilaterally in layer V of the AIC were caused by these confounders (see Table 2 B). In particular, with regard to the accentuated difference in fixation time between the groups compared, the analysis did not suggest that longer fixation was associated with lower GAD immunoreactivity.

**Table 2:** Analysis of confounding variables

**A** Intergroup comparisons						
			BV [cm^3^]	Age [years]	PMI [hours]	Fixation [days]
H (n = 13): median (q1, q3)			1475 (1446, 1543)	31 (25, 33)	43 (16, 81)	2926 (2373, 3815)
C (n = 12): median (q1, q3)			1367 (1278, 1422)	46 (39, 51)	27 (24, 46)	251 (180, 569)
Statistics:						
Test			*U*	*U*	*U*	*U*
Characteristic value			*Z* = − 3.079	*Z* = 2.64	*Z* = − 0.74	*Z* = − 3.657
*P* value			**0.0012**	**0.0066**	0.469	**0.000006**

**B** Correlation analysis between the numerical confounding variables listed above and the relative area of glutamic acid decarboxylase immunoreactive neuropil in the anterior insular cortex						
ROI	Group		BV	Age	PMI	Fixation
Layer III left	H	*r/P*	− 0.02/0.93	0.18/0.55	− 0.48/0.10	0.43/0.21
	C	*r/P*	0.48/0.12	− 0.53/0.08	0.30/0.34	0.41/0.18
Layer III right	H	*r/P*	0.10/0.75	0.15/0.62	0.12/0.70	0.51/0.13
	C	*r/P*	0.05/0.87	− 0.27/0.39	**0.74/0.01**	0.53/0.08
Layer V left	H	*r/P*	0.09/0.78	0.22/0.48	− 0.01/0.99	0.44/0.20
	C	*r/P*	− 0.06/0.85	− 0.09/0.77	− 0.08/0.80	0.17/0.58
Layer V right	H	*r/P*	0.14/0.65	**0.66/0.01**	0.07/0.0.82	0.38/0.28
	C	*r/P*	− 0.40/0.20	− 0.05/0.87	− 0.19/0.56	0.33/0.30

*H* heroin-addicted individuals, *C* controls, *n* number of cases, *q1 and q3* quartile 1 and 3, *BV* brain volume, *PMI* postmortem interval, *Fixation* fixation time, *ROI* region of interest, *r* correlation coefficient and *P P* value of the Spearman’s correlation (significant values are in bold)

### Correlation with previous volumetric findings

An additional correlation analysis was performed between GAD-ir neuropil density and AIC volume values, which were obtained previously [24]. This analysis revealed only a moderate negative correlation between cortical volume and layer V neuropil density in the right AIC in the control group. Furthermore, no correlations were found between current densitometric and previous volumetric results in both layers bilaterally in heroin-addicted individuals (non-significant Spearman’s correlation *P* values and irrelevant *r* values, see Table 3). Therefore, the correlation analysis did not suggest that the increase in GAD-ir neuropil density observed in this group was related to the previously observed decrease in AIC volume [24].

**Table 3:** Correlation analysis between the relative area of glutamic acid decarboxylase immunoreactive neuropil in layers III and V and the volume of the anterior insular cortex

Group		Layer III left	Layer III right	Layer V left	Layer V right
H	*r/P*	− 0.39/0.19	− 0.09/0.77	− 0.24/0.43	− 0.37/0.22
C	*r/P*	0.28/0.93	− 0.22/0.50	− 0.34/0.28	− **0.60/0.04**

*H* heroin-addicted individuals, *C* controls, *r* correlation coefficient and *P P* value of the Spearman’s correlation (significant values are in bold)

## Discussion

Our study suggests an impairment of the GABAergic system in the AIC in opioid addiction, as we found an increased density of GAD 65/67-ir neuropil in layers III and V in heroin-addicted males, with a significant increase in layer V bilaterally. The results do not seem to be confounded by significant differences in age, brain volume and fixation time existing between the groups compared. Furthermore, these findings cannot be explained by the significant volume reduction of the AIC in the addicted group found in our previous postmortem study [24].

According to experimental data, there is a close relationship between the level of GAD and the inhibitory activity of cortical interneurons [33], which is reflected at the synaptic level by GABAergic neuropil density [27, 28]. Therefore, our results suggest an increased GABAergic neurotransmission in the AIC in opioid addiction, which is accentuated in the deep cortical layer, whose pyramidal cells provide the main output to other cortical areas and subcortical structures involved in the addiction circuitry [2, 7]. Fast-spiking, parvalbumin-containing cortical GABAergic interneurons, known as basket cells, are the main source of the neuropil studied, which contacts the perisomatic region of layer III and V pyramidal cells [34]. Increased GABAergic inhibition of pyramidal cells in the AIC, which are involved in inhibitory control, may lead to the compulsive drug seeking observed in addicted humans and animal models of addiction [10, 35].

On the other hand, experimental data suggest that AIC interneurons may counteract pathological learning processes that reinforce the vicious cycle of addiction. Activation of GABA_B_ receptors, located predominantly on pyramidal cells in layer V of the AIC, prevents the negative affective learning induced by opioid withdrawal, which plays an important reinforcing role in drug-seeking behaviour [21]. Activation of these receptors by baclofen also prevents opioid-induced memory reconsolidation when applied in a vulnerable time window [22], similar to the activation of interneurons [23]. Therefore, these results suggest that the activation of the GABAergic system in the AIC during the early phase of addiction may counteract the further development of this process. However, it should be noted that the apparently contradictory results may be related to different functions provided by subsets of neuronal networks in the AIC [7, 10]. In addition, it is difficult to extrapolate the data from animal models to our findings in heroin-addicted individuals who died from overdose, most likely in the late stages of their disease.

As there is a lack of human postmortem research on the GABAergic system in the insula in addiction, not just opioid addiction, our results could only be compared with those obtained in neuroimaging studies. Experimental data suggest an inverse relationship between the GABA concentrations in brain regions measured by magnetic resonance spectroscopy and their resting-state activity in functional magnetic resonance imaging [36]. A recent human study also showed a negative correlation between task-induced cortical activity and GABA concentration [37]. Therefore, our data suggesting increased GABAergic activity in the AIC in opioid addiction may correspond to the decreased resting-state activity and glucose metabolism currently found in the AIC of heroin-addicted individuals [38].

The current results suggest that individuals addicted to opioids may benefit from downregulation of the hyperactive GABAergic system in the AIC. The ameliorative effect of this procedure has recently been demonstrated in an animal model of opioid addiction in the central nucleus of the amygdala, where the locally hyperactive GABAergic neurotransmission was normalised by the application of nociceptine [19]. Complementary to these results, attenuation of prefrontal interneuron hyperactivity by the dopamine D4 receptor agonist ameliorated impulsivity [39, 40], i.e. a behavioural endophenotype closely linked not only to addiction but also to suicide, in which addiction plays a prominent role according to epidemiological and clinical data [41, 42]. Furthermore, activation of the D4 receptor in an experimental model of opioid addiction has been shown to normalise elevated levels of GAD65/67 in striatal structures, including the globus pallidus [43], where we found elevated GAD65/67 in the internal part in heroin-addicted individuals [26]. Human data from cerebrovascular insults suggest additive effects on addiction disruption when striatal lesions are combined with insula lesions [10]. Therefore, simultaneous attenuation of hyperactive GABAergic function in different brain structures involved in the addiction circuitry, including the AIC, may be a plausible therapeutic option for the treatment of opioid use disorder in the future.

## Limitations

Our study is limited by several factors: (1) As with all postmortem analyses, our study is cross-sectional and longitudinal data are not available. (2) The cohort is small and we could only include brains from males because our brain bank does not contain postmortem brains from females addicted to heroin. (3) Due to limited clinical records, there are no reliable data on the duration of addiction or the amount of heroin used. Therefore, we cannot assess whether these factors influence our results. (4) The use of paraffin-embedded tissue is a limitation of our method compared to frozen brain samples, which would allow the application of a wider range of approaches. (5) Our method cannot distinguish between the populations of GABAergic interneurons in the AIC, which have different molecular and functional properties [32].

## Conclusion

Our results suggest a dysregulation of GABAergic activity in the AIC in heroin-addicted individuals, which may play a role in the dysfunction of this cortical region in opioid addiction. However, further research in larger cohorts using molecular techniques targeting the different populations of interneurons in the AIC is important to substantiate these findings.

## Supplementary Information

Below is the link to the electronic supplementary material.

Supplementary file1 (DOCX 29 KB)

## Data Availability

On behalf of all authors, the corresponding author states that the data being reported are accurate and are coming from the official source.

## References

[1] UNODC, World Drug Report (2023) (United Nations Publication, 2023). https://www.unodc.org/unodc/en/data-and-analysis/world-drug-report-2023.html

[2] Naqvi NH, Bechara A (2010) The insula and drug addiction: an interoceptive view of pleasure, urges, and decision-making. Brain Struct Funct 214:435–450. 10.1007/s00429-010-0268-7 20512364 10.1007/s00429-010-0268-7 PMC3698865

[3] Nieuwenhuys R (2012) The insular cortex: a review. Prog Brain Res 195:123–163. 10.1016/B978-0-444-53860-4.00007-6 22230626 10.1016/B978-0-444-53860-4.00007-6

[4] Fermin ASR, Friston K, Yamawaki S (2022) An insula hierarchical network architecture for active interoceptive inference. R Soc Open Sci 9:220226. 10.1098/rsos.220226 35774133 10.1098/rsos.220226 PMC9240682

[5] Koob GF (2021) Drug addiction: hyperkatifeia/negative reinforcement as a framework for medications development. Pharmacol Rev 73:163–201. 10.1124/pharmrev.120.000083 33318153 10.1124/pharmrev.120.000083 PMC7770492

[6] Koob GF, Volkow ND (2016) Neurobiology of addiction: a neurocircuitry analysis. Lancet Psychiatry 3:760–773. 10.1016/S2215-0366(16)00104-8 27475769 10.1016/S2215-0366(16)00104-8 PMC6135092

[7] Ibrahim C, Rubin-Kahana DS, Pushparaj A, Musiol M, Blumberger DM, Daskalakis ZJ, Zangen A, Le Foll B (2019) The insula: a brain stimulation target for the treatment of addiction. Front Pharmacol 10:720. 10.3389/fphar.2019.00720 31312138 10.3389/fphar.2019.00720 PMC6614510

[8] Ghaziri J, Tucholka A, Girard G, Houde JC, Boucher O, Gilbert G, Descoteaux M, Lippé S, Rainville P, Nguyen DK (2017) The corticocortical structural connectivity of the human insula. Cereb Cortex 27:1216–1228. 10.1093/cercor/bhv308 26683170 10.1093/cercor/bhv308

[9] Ghaziri J, Tucholka A, Girard G, Boucher O, Houde JC, Descoteaux M, Obaid S, Gilbert G, Rouleau I, Nguyen DK (2018) Subcortical structural connectivity of insular subregions. Sci Rep 8:8596. 10.1038/s41598-018-26995-0 29872212 10.1038/s41598-018-26995-0 PMC5988839

[10] Naqvi NH, Gaznick N, Tranel D, Bechara A (2014) The insula: a critical neural substrate for craving and drug seeking under conflict and risk. Ann N Y Acad Sci 1316:53–70. 10.1111/nyas.12415 24690001 10.1111/nyas.12415 PMC4114146

[11] Moeller SJ, London ED, Northoff G (2016) Neuroimaging markers of glutamatergic and GABAergic systems in drug addiction: relationships to resting-state functional connectivity. Neurosci Biobehav Rev 61:35–52. 10.1016/j.neubiorev.2015.11.010 26657968 10.1016/j.neubiorev.2015.11.010 PMC4731270

[12] Shyu C, Chavez S, Boileau I, Le Foll B (2022) Quantifying GABA in addiction: a review of proton magnetic resonance spectroscopy studies. Brain Sci 12:918. 10.3390/brainsci12070918 35884725 10.3390/brainsci12070918 PMC9316447

[13] Laprade N, Soghomonian JJ (1999) Gene expression of the GAD67 and GAD65 isoforms of glutamate decarboxylase is differentially altered in subpopulations of striatal neurons in adult rats lesioned with 6-OHDA as neonates. Synapse 33:36–48 10380849 10.1002/(SICI)1098-2396(199907)33:1<36::AID-SYN4>3.0.CO;2-0

[14] Wei J, Wu JY (2008) Post-translational regulation of L-glutamic acid decarboxylase in the brain. Neurochem Res 33:1459–1465. 10.1007/s11064-008-9600-5 18270816 10.1007/s11064-008-9600-5

[15] Kanold PO, Shatz CJ (2006) Subplate neurons regulate maturation of cortical inhibition and outcome of ocular dominance plasticity. Neuron 51:627–638. 10.1016/j.neuron.2006.07.008 16950160 10.1016/j.neuron.2006.07.008

[16] Jagasia R, Steib K, Englberger E, Herold S, Faus-Kessler T, Saxe M, Gage FH, Song H, Lie DC (2009) GABA-cAMP response element-binding protein signaling regulates maturation and survival of newly generated neurons in the adult hippocampus. J Neurosci 29:7966–7977. 10.1523/JNEUROSCI.1054-09.2009 19553437 10.1523/JNEUROSCI.1054-09.2009 PMC2776747

[17] Gonzalez-Burgos G, Cho RY, Lewis DA (2015) Alterations in cortical network oscillations and parvalbumin neurons in schizophrenia. Biol Psychiatry 77:1031–1040. 10.1016/j.biopsych.2015.03.010 25863358 10.1016/j.biopsych.2015.03.010 PMC4444373

[18] Luster BR, Cogan ES, Schmidt KT, Pati D, Pina MM, Dange K, McElligott ZA (2020) Inhibitory transmission in the bed nucleus of the stria terminalis in male and female mice following morphine withdrawal. Addict Biol 25:e12748. 10.1111/adb.12748 30963693 10.1111/adb.12748 PMC6785353

[19] Kallupi M, Carrette LLG, Kononoff J, Solberg Woods LC, Palmer AA, Schweitzer P, George O, de Guglielmo G (2020) Nociceptin attenuates the escalation of oxycodone self-administration by normalizing CeA-GABA transmission in highly addicted rats. Proc Natl Acad Sci U S A 117:2140–2148. 10.1073/pnas.1915143117 31932450 10.1073/pnas.1915143117 PMC6994987

[20] Bayer R, Franke H, Ficker C, Richter M, Lessig R, Büttner A, Weber M (2015) Alterations of neuronal precursor cells in stages of human adult neurogenesis in heroin addicts. Drug Alcohol Depend 156:139–149. 10.1016/j.drugalcdep.2015.09.005 26416695 10.1016/j.drugalcdep.2015.09.005

[21] Li CL, Zhu N, Meng XL, Li YH, Sui N (2013) Effects of inactivating the agranular or granular insular cortex on the acquisition of the morphine-induced conditioned place preference and naloxone-precipitated conditioned place aversion in rats. J Psychopharmacol 27:837–844. 10.1177/0269881113492028 23784741 10.1177/0269881113492028

[22] Sun K, Mu Q, Chang H, Zhang C, Wang Y, Rong S, Liu S, Zuo D, He Z, Wan D, Yang H, Wang F, Sun T (2020) Postretrieval microinjection of baclofen into the agranular insular cortex inhibits morphine-induced CPP by disrupting reconsolidation. Front Pharmacol 11:743. 10.3389/fphar.2020.00743 32508658 10.3389/fphar.2020.00743 PMC7248341

[23] Sun K, Xiao L, Wu Y, Zuo D, Zhang C, Liu S, He Z, Rong S, Wang F, Sun T (2020) GABAergic neurons in the insular cortex play an important role in cue-morphine reward memory reconsolidation. Life Sci 254:117655. 10.1016/j.lfs.2020.117655 32277980 10.1016/j.lfs.2020.117655

[24] Müller UJ, Schmalenbach LJ, Dobrowolny H, Guest PC, Schlaaff K, Mawrin C, Truebner K, Bogerts B, Gos T, Bernstein HG, Steiner J (2023) Reduced anterior insular cortex volume in male heroin addicts: a postmortem study. Eur Arch Psychiatry Clin Neurosci 273:1233–1241. 10.1007/s00406-023-01553-6 36719479 10.1007/s00406-023-01553-6 PMC9888352

[25] Müller UJ, Mawrin C, Frodl T, Dobrowolny H, Busse S, Bernstein HG, Bogerts B, Truebner K, Steiner J (2019) Reduced volumes of the external and internal globus pallidus in male heroin addicts: a postmortem study. Eur Arch Psychiatry Clin Neurosci 269:317–324. 10.1007/s00406-018-0939-6 30173319 10.1007/s00406-018-0939-6

[26] Gos A, Steiner J, Trübner K, Ungewickell J, Mawrin C, Karnecki K, Kaliszan M, Gos T (2024) Inverse pattern of GABAergic system impairment in the external versus internal globus pallidus in male heroin addicts. Eur Arch Psychiatry Clin Neurosci 274:445–452. 10.1007/s00406-023-01656-0 37507486 10.1007/s00406-023-01656-0 PMC10914887

[27] Gu F, Parada I, Shen F, Li J, Bacci A, Graber K, Taghavi RM, Scalise K, Schwartzkroin P, Wenzel J, Prince DA (2017) Structural alterations in fast-spiking GABAergic interneurons in a model of posttraumatic neocortical epileptogenesis. Neurobiol Dis 108:100–114. 10.1016/j.nbd.2017.08.008 28823934 10.1016/j.nbd.2017.08.008 PMC5927780

[28] Bijlsma A, Omrani A, Spoelder M, Verharen JPH, Bauer L, Cornelis C, de Zwart B, van Dorland R, Vanderschuren LJMJ, Wierenga CJ (2022) Social play behavior is critical for the development of prefrontal inhibitory synapses and cognitive flexibility in rats. J Neurosci 42:8716–8728. 10.1523/JNEUROSCI.0524-22.2022 36253083 10.1523/JNEUROSCI.0524-22.2022 PMC9671579

[29] Gos T, Günther K, Bielau H, Dobrowolny H, Mawrin C, Trübner K, Brisch R, Steiner J, Bernstein HG, Jankowski Z, Bogerts B (2009) Suicide and depression in the quantitative analysis of glutamic acid decarboxylase-Immunoreactive neuropil. J Affect Disord 113:45–55. 10.1016/j.jad.2008.04.021 18538859 10.1016/j.jad.2008.04.021

[30] Gos T, Steiner J, Bielau H, Dobrowolny H, Günther K, Mawrin C, Krzyżanowski M, Hauser R, Brisch R, Bernstein HG, Jankowski Z, Braun K, Bogerts B (2012) Differences between unipolar and bipolar I depression in the quantitative analysis of glutamic acid decarboxylase-immunoreactive neuropil. Eur Arch Psychiatry Clin Neurosci 262:647–655. 10.1007/s00406-012-0315-x 22526728 10.1007/s00406-012-0315-x PMC3491185

[31] Steiner J, Brisch R, Schiltz K, Dobrowolny H, Mawrin C, Krzyżanowska M, Bernstein HG, Jankowski Z, Braun K, Schmitt A, Bogerts B, Gos T (2016) GABAergic system impairment in the hippocampus and superior temporal gyrus of patients with paranoid schizophrenia: a post-mortem study. Schizophr Res 177:10–17. 10.1016/j.schres.2016.02.018 26922657 10.1016/j.schres.2016.02.018

[32] Türe U, Yaşargil DC, Al-Mefty O, Yaşargil MG (1999) Topographic anatomy of the insular region. J Neurosurg 90:720–733. 10.3171/jns.1999.90.4.0720 10193618 10.3171/jns.1999.90.4.0720

[33] Lazarus MS, Krishnan K, Huang ZJ (2015) GAD67 deficiency in parvalbumin interneurons produces deficits in inhibitory transmission and network disinhibition in mouse prefrontal cortex. Cereb Cortex 25:1290–1296. 10.1093/cercor/bht322 24275833 10.1093/cercor/bht322 PMC4481616

[34] Tremblay R, Lee S, Rudy B (2016) GABAergic interneurons in the neocortex: from cellular properties to circuits. Neuron 91:260–292. 10.1016/j.neuron.2016.06.033 27477017 10.1016/j.neuron.2016.06.033 PMC4980915

[35] Chen H, Lasek AW (2020) Perineuronal nets in the insula regulate aversion-resistant alcohol drinking. Addict Biol 25:e12821. 10.1111/adb.12821 31433552 10.1111/adb.12821 PMC7032993

[36] Alcaro A, Panksepp J, Witczak J, Hayes DJ, Northoff G (2010) Is subcortical-cortical midline activity in depression mediated by glutamate and GABA? a cross-species translational approach. Neurosci Biobehav Rev 34:592–605. 10.1016/j.neubiorev.2009.11.023 19958790 10.1016/j.neubiorev.2009.11.023

[37] Koush Y, de Graaf RA, Kupers R, Dricot L, Ptito M, Behar KL, Rothman DL, Hyder F (2021) Metabolic underpinnings of activated and deactivated cortical areas in human brain. J Cereb Blood Flow Metab 41:986–1000. 10.1177/0271678X21989186 33472521 10.1177/0271678X21989186 PMC8054719

[38] Jin L, Yuan M, Chen J, Zhang W, Wang L, Wei Y, Li Y, Guo Z, Wang W, Wei L, Li Q (2023) Abnormal cerebral metabolism and metabolic connectivity in individuals with heroin dependence: an integrated resting-state PET/fMRI study in large-scale networks. J Psychiatry Neurosci 48:E295–E304. 10.1503/jpn.220171 37437921 10.1503/jpn.220171 PMC10355996

[39] Hayes DJ, Jupp B, Sawiak SJ, Merlo E, Caprioli D, Dalley JW (2014) Brain γ-aminobutyric acid: a neglected role in impulsivity. Eur J Neurosci 39:1921–1932. 10.1111/ejn.12485 24460847 10.1111/ejn.12485

[40] Yan Z, Rein B (2022) Mechanisms of synaptic transmission dysregulation in the prefrontal cortex: pathophysiological implications. Mol Psychiatry 27:445–465. 10.1038/s41380-021-01092-3 33875802 10.1038/s41380-021-01092-3 PMC8523584

[41] Karnecki K, Gos T, Steiner J, Mańkowski D, Kaliszan M (2023) Epidemiology of suicide in the Tri-City metropolitan area in Poland in 2010–2019. Eur Arch Psychiatry Clin Neurosci 273:911–920. 10.1007/s00406-022-01548-9 36583739 10.1007/s00406-022-01548-9 PMC10238313

[42] Rizk MM, Herzog S, Dugad S, Stanley B, Suicide Risk and Addiction (2021) The impact of alcohol and opioid use disorders. Curr Addict Rep 8:194–207. 10.1007/s40429-021-00361-z 33747710 10.1007/s40429-021-00361-z PMC7955902

[43] Negrete-Díaz JV, Shumilov K, Real MÁ, Medina-Luque J, Valderrama-Carvajal A, Flores G, Rodríguez-Moreno A, Rivera A (2019) Pharmacological activation of dopamine D_4_ receptor modulates morphine-induced changes in the expression of GAD_65/67_ and GABA_B_ receptors in the basal ganglia. Neuropharmacology 152:22–29. 10.1016/j.neuropharm.2019.01.024 30682345 10.1016/j.neuropharm.2019.01.024

