# Supplementary material for: Impairment of the GABAergic system in the anterior insular cortex of heroin-addicted males
Source: Eur Arch Psychiatry Clin Neurosci. 2024 Jul 9;275(1):219–26. doi: 10.1007/s00406-024-01848-2 (PMC11799008; doi:10.1007/s00406-024-01848-2)
Supplement: Supplementary file 1 — Supplementary file1 (DOCX 29 KB) [file 406_2024_1848_MOESM1_ESM.docx]

**Impairment of the GABAergic system in the anterior insular cortex of heroin-addicted males**

Anna Gos, Johann Steiner, Kurt Trübner, Christian Mawrin, Michał Kaliszan, Tomasz Gos

**European Archives of Psychiatry and Clinical Neuroscience**

Corresponding author:

Tomasz Gos, MD, PhD

Department of Forensic Medicine

Medical University of Gdańsk

ul. Dębowa 23

80-204 Gdańsk, Poland

E-mail: [gost@gumed.edu.pl](mailto:gost@gumed.edu.pl)

**Supplementary Table** Diagnostic and demographic data, and the values of the relative density of glutamic acid decarboxylase immunoreactive neuropil in percent (GAD-ir neuropil rel. density [%]) in the layers III and V of the anterior insular cortex bilaterally in controls (n=12) and heroin-addicted male subjects (n=13). *Abbreviations:* BV – brain volume; PMI – postmortem interval; Fixation – fixation time; *q1* and *q3* – quartile 1 and 3. (Significant U-test *P* values are in bold.)

| Case ID |  | BV  [cm^3^] | Age [years] | PMI [hours] | Fixation [days] | Layer III left  GAD-ir neuropil  rel. density [%] | Layer V left  GAD-ir neuropil  rel. density [%] | Layer III right  GAD-ir neuropil  rel. density [%] | Layer V right  GAD-ir neuropil  rel. density [%] |
| --- | --- | --- | --- | --- | --- | --- | --- | --- | --- |
|  |  |  |  |  |  |  |  |  |  |
|  | **Controls: cause of death** |  |  |  |  |  |  |  |  |
| 1 | Acute myocardial infarction | 1398 | 47 | 24 | 179 | 0.90 | 1.29 | 0.85 | 2.25 |
| 2 | Acute respiratory failure (aspiration) | 1157 | 47 | 24 | 84 | 1.35 | 1.71 | 1.69 | 2.45 |
| 3 | Sudden cardiac death | 1398 | 56 | 30 | 225 | 2.91 | 1.75 | 2.46 | 2.33 |
| 4 | Acute myocardial infarction | 1495 | 38 | 19 | 70 | 2.12 | 2.28 | 1.70 | 1.90 |
| 5 | Acute myocardial infarction | 1495 | 40 | 96 | 180 | 1.64 | 1.23 | 1.88 | 1.20 |
| 6 | Ruptured aortic aneurysm | 1263 | 64 | 35 | 240 | 1.07 | 1.94 | 2.45 | 1.59 |
| 7 | Acute respiratory failure (pneumonia) | 1354 | 39 | 4 | 330 | 1.55 | 1.30 | 1.29 | 1.99 |
| 8 | Pulmonary embolism (acute cor pulmonale) | 1379 | 54 | 24 | 250 | 1.48 | 1.68 | 1.46 | 1.47 |
| 9 | Sudden cardiac death | 1249 | 46 | 24 | 290 | 1.51 | 1.94 | 1.63 | 2.99 |
| 10 | Accidental strangulation (autoerotic death) | 1302 | 45 | 44 | 1603 | 1.82 | 2.25 | 3.46 | 2.89 |
| 11 | Hypovolaemic shock | 1446 | 28 | 48 | 1043 | 2.37 | 2.21 | 2.93 | 2.35 |
| 12 | Acute myocardial infarction | 1292 | 29 | 60 | 808 | 2.05 | 1.38 | 2.87 | 1.69 |
|  | *Controls: median (q1, q3)* | *1367 (1278, 1422)* | *46 (39, 51)* | *27 (24, 46)* | *251 (180, 569)* | *1.59 (1.42, 2.09)* | *1.73 (1.34, 2.08)* | *1.79 (1.55, 2.67)* | *2.12 (1.64, 2.40)* |
|  |  |  |  |  |  |  |  |  |  |
|  | **Heroin-addicted individuals: cause of death and** **substances used in addition to heroin** |  |  |  |  |  |  |  |  |
| 13 | Heroin overdose; none | 1446 | 25 | 30 | 1426 | 2.50 | 1.37 | 1.62 | 2.48 |
| 14 | Heroin overdose; cannabis, barbiturates, benzodiazepines, codeine | 1427 | 33 | 85 | 2185 | 1.73 | 1.50 | 2.10 | 3.23 |
| 15 | Heroin overdose; cannabis | 1475 | 24 | 49 | 2373 | 2.83 | 2.53 | 2.23 | 1.34 |
| 16 | Heroin overdose; alcohol, cannabis, cocaine, codeine, benzodiazepines, barbiturates | 1475 | 31 | 10 | 2914 | 2.98 | 2.22 | 1.76 | 2.02 |
| 17 | Heroin overdose; alcohol, hallucinogens, cocaine, barbiturates | 1562 | 40 | 16 | 2938 | 1.97 | 3.68 | 4.02 | 3.77 |
| 18 | Heroin overdose; none | 1398 | 21 | 164 | 2702 | 1.22 | 3.12 | 3.52 | 1.66 |
| 19 | Heroin overdose; benzodiazepines, barbiturates | 1543 | 32 | 16 | 3202 | 2.67 | 2.13 | 3.40 | 2.51 |
| 20 | Heroin overdose; morphine | 1446 | 47 | 96 | - | 3.44 | 2.63 | 3.39 | 2.84 |
| 21 | Heroin overdose; none | 1543 | 40 | 81 | - | 0.97 | 2.04 | 2.29 | 2.92 |
| 22 | Heroin overdose; none | 1456 | 30 | 63 | - | 1.34 | 2.56 | 3.76 | 3.00 |
| 23 | Heroin overdose; unknown | 1408 | 32 | 16 | 3906 | 3.87 | 3.21 | 2.73 | 2.86 |
| 24 | Heroin overdose; unknown | 1581 | 31 | 43 | 3851 | 2.83 | 3.71 | 2.38 | 2.77 |
| 25 | Heroin overdose; unknown | 1533 | 21 | 32 | 3815 | 1.85 | 1.28 | 2.38 | 2.77 |
|  | *Heroin-addicted: median (q1, q3)* | *1475 (1446, 1543)* | *31 (25, 33)* | *43 (16, 81)* | *2926 (2373, 3815)* | *2.50 (1.73, 2.83)* | *2.53 (2.04, 3.12)* | *2.38 (2.23, 3.40)* | *2.77 (2.48, 2.92)* |
|  | **Statistics** |  |  |  |  |  |  |  |  |
|  | test | *U* | *U* | *U* | *U* | *U* | *U* | *U* | *U* |
|  | Characteristic value | *Z* = −3.080 | *Z* = 2.64 | *Z* = −0.737 | *Z =* −3.857 | *Z= −*1.605 | *Z* = −2.258 | *Z = −*1.768 | *Z* = −2.095 |
|  | *P* value | **0.0012** | **0.0066** | 0.470 | **0.000006** | 0.110 | **0.022** | 0.077 | **0.035** |
